# Adaptation to soil type contributes little to local adaptation in an Italian and a Swedish ecotype of *Arabidopsis thaliana* on contrasting soils

**DOI:** 10.1098/rsbl.2024.0236

**Published:** 2024-09-11

**Authors:** Thomas James Ellis, Jon Ågren

**Affiliations:** ^1^ Plant Ecology and Evolution, Department of Ecology and Genetics, EBC, Uppsala University, Uppsala, Sweden; ^2^ Gregor Mendel Institute of Molecular Plant Sciences, Austrian Academy of Sciences, Doktor-Bohr-Gasse 3, Vienna 1010, Austria

**Keywords:** local adaptation, edaphic, *Arabidopsis*, soil

## Abstract

Natural populations are subject to selection caused by a range of biotic and abiotic factors in their native habitats. Identifying these agents of selection and quantifying their effects is key to understanding how populations adapt to local conditions. We performed a factorial reciprocal-transplant experiment using locally adapted ecotypes of *Arabidopsis thaliana* at their native sites to distinguish the contributions of adaptation to soil type and climate. Overall adaptive differentiation was strong at both sites. However, we found only very small differences in the strength of selection on local and non-local soil, and adaptation to soil type at most constituted only a few per cent of overall adaptive differentiation. These results indicate that local climatic conditions rather than soil type are the primary driver of adaptive differentiation between these ecotypes.

## 1. Introduction

Reciprocal transplant experiments have demonstrated local adaptation across several spatial scales and in many species [1,2], but in many cases, the agents responsible for divergent selection have not been established. This is problematic as a full understanding of the ecological causes of adaptation requires that both adaptive traits and agents of selection are identified. Terrestrial plants derive nutrients required for growth predominantly through interactions with the soil and soil biota. Since the biotic and abiotic characteristics of soil vary immensely, these interactions can select for plant traits that promote increased performance on a specific soil type. Many studies have characterized plant adaptation to strongly stressful soil conditions, such serpentine soils, soils with high concentrations of heavy metals and with high salinity [3–7]. However, subtle differences in soil composition can also be sufficient to result in divergent selection on plants [8–11]. In this way, soil type may act as a selective agent contributing to local adaptation.

In this study, we investigate adaptation to native soils in two locally adapted ecotypes of *Arabidopsis thaliana* in Italy and Sweden. The native sites of these ecotypes differ strongly in climate [12], but also in soil composition. The acidic soil at the Swedish site has a higher iron content, and lower availability of magnesium and calcium compared to the slightly alkaline soil at the Italian site [13]. Previous work using reciprocal transplant experiments of the local ecotypes and recombinant inbred lines derived from them have demonstrated local adaptation to climatic conditions [12,14–18]. However, neither a field experiment at the native sites in Italy and Sweden using the parental ecotypes grown on soil from both source sites, nor a growth-chamber experiment that disentangled the effects of biotic and abiotic components of these soils found evidence for adaptation of plant ecotypes to their native soils [13,19]. One caveat is that these studies were limited by relatively small sample sizes and substantial block effects. Moreover, the field experiment was conducted in a single year, and no plants of the Italian ecotype survived to reproduce at the Swedish site, making it difficult to fully evaluate the effect of soil on adaptive differentiation. As such, it is difficult to conclusively rule out a role for adaptation to soil type in local adaptation between these ecotypes.

We conducted a field experiment in which plant ecotypes and soil were reciprocally transplanted between the native sites of the two *A. thaliana* ecotypes to disentangle the contributions of adaptation to soil type and climate. We employ a design that builds on the weaknesses of previous studies by using a fully randomized design with substantially larger sample sizes than those used in earlier work [13,19]. We tested the prediction that if soil type contributes to local adaptation, the fitness advantage of local plants will be greater when grown on the local soil than on the non-local soil.

## 2. Methods

We compared plant fitness in a fully crossed reciprocal-transplant experiment in which site, soil type and plant ecotype were varied. We grew local ecotypes of *A. thaliana* at sites in central Italy (Castelnuovo) and north-central Sweden (Rödåsen) on soils collected at each site. Sites, ecotypes used and experimental set-up followed those of [12]. Genetic variation within sites is small [14], especially in comparison to variation between sites, so following previous work we used one representative local genotype at each site which were previously used as the parents of a RIL mapping population [12,14,16,17,20].

We collected soil from the two source sites in spring 2017, and stored this in plastic buckets at 6°C. At both sites, we collected the 5–10 cm of soil closest to the surface. At the Swedish site, we collected soil in patches which at the time had closed vegetation with no observable *A. thaliana*. In Italy, we collected soil from a vertical wall of soil with no established *A. thaliana*. Germination from soil collected this way and used in field experiments has been found to be very low [16,21].

At these sites, *A. thaliana* is a winter annual. To ensure that the experimental plants experienced similar conditions as plants in the local population, we timed the beginning and end of the field experiments to correspond to times when local plants naturally germinate and senesce. We germinated seeds of each ecotype on agar in Petri dishes in a growth room at Uppsala University, transported these to field sites and transplanted seedlings to 299 cell plug trays with individual cells of 20 mm × 20 mm × 40 mm (HerkuPlast Kubern GmbH, Ering, Germany). We transplanted 2080 seedlings across the two sites, corresponding to 256 and 264 of the local and non-local ecotype, respectively, on each soil type at each site on 7 September 2017 in Sweden and on 9 November 2017 in Italy. The difference in sample size was to compensate somewhat for the typically lower winter survival of non-local ecotypes, which reduces the precision of fecundity estimates. We filled the lowest three rows of the plug trays (39 positions) with additional plants to reduce edge effects. On 23 April 2018 in Italy and on 17 June 2018 in Sweden, we recorded survival to reproduction, and fruit number of each reproductive plant in non-edge rows. For 707 plants in Italy and 488 plants in Sweden that still had at least one intact mature silique at the time of harvest, we estimated seed number per fruit by counting all viable seeds from one mature unopened fruit as described by [17]. We estimated seed number per planted seedling, a proxy for overall fitness, as the product of fruit number and site–soil–ecotype–mean seed number per fruit.

For each site–soil combination, we estimated selection based on ecotype–mean survival, fecundity and overall fitness, respectively. We quantified selection against the non-local ecotype as *s* = 1− (*w*_non-local_/*w*_local_), where *w*_local_ and *w*_non-local_ are the mean absolute fitness values for the local and non-local ecotypes, respectively. We quantified 95% CIs around fitness and selection estimates by drawing 1000 bootstrap samples of the data stratified by site–soil–ecotype treatment, and recalculating parameters for each. We tested the null hypothesis that there is no difference in selection between the two soil types by permuting soil types within sites and recalculating selection coefficients. We calculated *p* values as the proportion of permuted datasets for which the difference in selection between the two soil types at each site was greater than in the observed data. We multiplied this proportion by two to make this a two-sided test.

We performed statistical analyses in RStudio 2023.06.1+524 using R 4.2.1 [22,23].

## 3. Results

**Figure 1.**
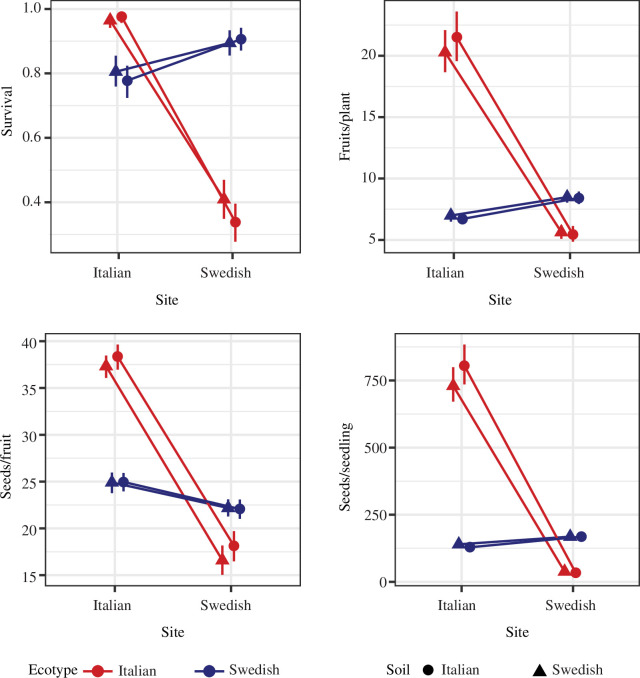
Fitness and its components for Italian and Swedish ecotypes when growing at the Italian and Swedish sites, on soils collected at each of the two sites. Plots show means ± 95% CI for survival to reproduction, number of fruits per reproductive plant, number of seeds per fruit and number of seeds per planted seedling.

**Figure 2.**
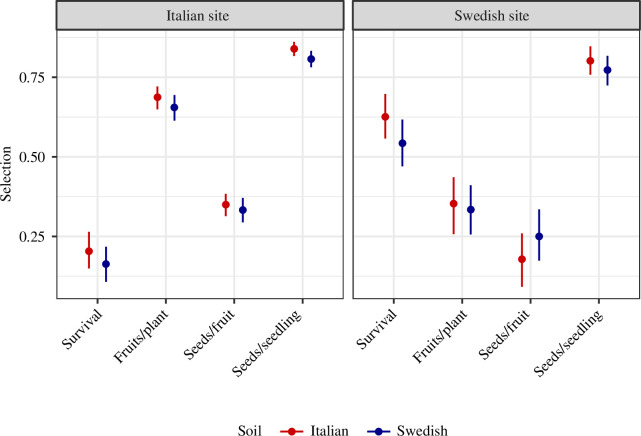
Selection favouring the local ecotype on Italian and Swedish soils at the Italian and Swedish sites. Plots show selection coefficients with 95% CIs for survival to reproduction, number of fruits per reproductive plant, number of seeds per fruit and number of seeds per planted seedling.

Overall adaptive differentiation on local soils was strong at both sites (figure 1). Local ecotypes showed 4.4- and 6.2-fold higher overall fitness (seed number per planted seedling) on local soils in Italy and Sweden, respectively. Local ecotypes also showed higher fitness through all three components of fitness, although selection through survival was stronger in Sweden, and selection through fecundity components stronger in Italy (figure 2). The patterns of selection found in this study are consistent with previous studies of the same ecotypes [12,14,17].

We found only small differences in the strength of selection on the two soil types (figure 2). At the Italian site, selection quantified based on overall fitness was slightly stronger on the local soil ( Δs = 0.032), and this difference approached, but did not exceed, the threshold for statistical significance (*p* = 0.062). Similarly, estimates of selection based on each of the three fitness components were slightly larger on the local soil than on the non-local soil, but these differences were not statistically significant (*p* ≥ 0.206). At the Swedish site, the estimate of selection through number of seeds per fruit was slightly larger on the local than on the non-local soil ( Δs = 0.071), but the opposite was true for estimates of selection through survival, number of fruits per reproductive plant and overall fitness ( Δs = 0.084, 0.019, 0.029, respectively), although none of these differences were statistically significant (*p* ≥ 0.092). These results indicate that there were no large differences in the strength of selection between soil types at either site.

## 4. Discussion

This study quantified the contribution of adaptation to soil type to overall adaptive differentiation between two locally adapted ecotypes of *A. thaliana* occurring on contrasting soils and close to the northern and southern range limit in Europe. Although overall fitness differences between local and non-local ecotypes were very strong at both sites, differences in the strength of selection when measured on local and non-local soil were small and not convincingly different from zero. These observations suggest that adaptation to local soil type makes at most a very limited contribution to adaptive differentiation between these ecotypes.

The results are unlikely to be an artefact of low statistical power. Sample sizes for each site–soil–ecotype treatment were large, and the confidence intervals around estimates of fitness and selection coefficients were narrow (figures 1 and 2). It is plausible that an even larger experiment could have detected a statistically significant difference in the strength of selection between soil types, especially for overall fitness in Italy. However, the absolute magnitude of the difference would remain very small, and as such the biological significance of the result would remain slight. The main conclusion that adaptation to soil type contributes little to local adaptation between these ecotypes would thus remain unchanged.

The plug trays used in the present study have open bottoms which could potentially affect the results. The open bottoms are necessary for drainage, but this means that if a plant grows sufficiently large, its roots can potentially grow through the plug into the underlying soil, which would weaken any effect of the soil treatment. However, in the present experiment, plants were small and of similar size to naturally occurring plants at the study sites, and roots did not reach through the plugs. Moreover, although the contact between experimental soil and underlying soil through the small hole at the bottom of each plug might have reduced differences between soil treatments, this effect is likely to have been weak.

The absence of evidence for adaptation to local soil suggests that the two ecotypes have broad tolerance to soil composition. This is in contrast to reports of adaptation to calcareous and high-salinity soils in *A. thaliana* in northeastern Spain [5,24,25]. The soils at our study sites do not differ in sodium or carbonate concentrations [19]. However, differences in pH, which strongly affect mineral uptake and use [26], and in magnesium, calcium and iron concentrations were among the largest recorded among 17 *A*. *thaliana* sites sampled across Europe [13], and in a sample of 168 populations in southern France [27]. Further studies should determine whether lack of adaptive differentiation in response to these aspects of soil composition is a general phenomenon in *A. thaliana*, and whether a broad tolerance to soil conditions provides an advantage in this opportunistic colonizer of disturbed habitats.

Previous work suggests that adaptation to local climatic conditions is the primary driver of adaptive differentiation between the two ecotypes examined in this study. Differences in seed dormancy, and phenology of seed germination and flowering are consistent with documented divergent selection on these traits, and with differences between the native sites in the timing of conditions favourable for seedling establishment, growth and seed production [16,20,28]. Moreover, in Sweden, minimum winter temperature is a strong predictor of strength of selection against the non-local ecotype [12], and local plants show higher freezing tolerance and ability to photosynthesize at low temperatures compared to the Italian ecotype [15,18,29]. Differences between the two ecotypes in seed dormancy, flowering time and cold tolerance are consistent with large-scale latitudinal variation in these traits across the European range of *A. thaliana* [21,30–33]. However, additional field experiments will be required to determine to what extent variation in these traits reflects divergent selection among local ecotypes, gene flow and historical factors across a variety of spatial scales.

Evidence for adaptation to climate has been reported in a large number of plant species with a wide distribution [2], but studies of the present kind, in which reciprocal transplants across climatic gradients have been combined with manipulation of soil conditions are still rare (e.g. [9]). As a result, the relative importance of adaptation to climate and to soil is still incompletely known. We suggest that this is an important gap to fill for understanding and predicting the consequences of environmental change for species distributions, local adaptation and the management of declining species.

## Data Availability

Data and code to reproduce the results in this manuscript are available on GitHub [34] and Zenodo [35].
